# Identification and Validation of Three m6A Regulators: *FTO*, *HNRNPC*, and *HNRNPA2B1* as Potential Biomarkers for Endometriosis

**DOI:** 10.3390/genes14010086

**Published:** 2022-12-28

**Authors:** Jiani Sun, Lei Gan, Jing Sun

**Affiliations:** 1 Shanghai Key Laboratory of Maternal Fetal Medicine, Shanghai Institute of Maternal-Fetal Medicine and Gynecologic Oncology, Shanghai First Maternity and Infant Hospital, School of Medicine, Tongji University, Shanghai 200092, China; 2 Department of Gynaecology and Obstetrics, Ningbo First Hospital, Ningbo 315010, China

**Keywords:** endometriosis, m6A RNA methylation regulators, random forest, consensus clustering, immune infiltration, pyroptosis, inflammation

## Abstract

Background: N6-methyladenosine is involved in numerous biological processes. However, the significance of m6A regulators in endometriosis is still unclear. Methods: We extracted three significant m6A regulators between non-endometriosis and endometriosis patients from GSE6364 and then we used the random forest model to obtain significant m6A regulators. In addition, we used the nomogram model to evaluate the prevalence of endometriosis. The predictive ability of the candidate genes was evaluated through the receiver operating characteristic curves, while the expression of candidate biomarkers was validated via Western blotting. Additionally, according to candidate genes, we identified m6A subtypes based on which functional enrichment analysis and immune infiltration were performed. Results: Three significant m6A regulators (fat mass and obesity-associated protein, heterogeneous nuclear ribonucleoprotein A2/B1, and heterogeneous nuclear ribonucleoprotein C) were discovered. We identified three m6A subtypes, including clusterA, clusterB, and clusterC. ClusterB was demonstrated to be correlated with significantly overexpressed *VEGF* and notably downregulated *ESR1* and *PGR*, which are convincing biomarkers of endometriosis. Furthermore, we discovered that patients in clusterB were associated with high levels of neutrophil infiltration, a reduced Treg/Th17 ratio, and overexpressed pyroptosis-related genes, which also indicated that clusterB was highly linked to endometriosis. Conclusion: In conclusion, m6A regulators are of great significance for the occurrence and process of endometriosis. The findings of our study provide novel insights into the underlying molecular mechanism of endometriosis. The novel investigation of m6A patterns and their correlation with immunity may also help to guide the clinical diagnosis, provide prognostic significance, and develop immunotherapy strategies for endometriosis patients.

## 1. Introduction

Endometriosis, a condition where the endometrium appears outside the uterus, mainly in the pelvic peritoneum and ovaries, is a common and complex gynecological disease [1]. Patients with endometriosis may suffer from chronic pelvic pain, dysmenorrhea, and infertility, all of which seriously affect their daily life. Additionally, these non-specific symptoms make endometriosis difficult to recognize, especially at the early stages of the disease [2]. The average interval from the emergence of symptoms to diagnosis is five to ten years [3,4]. Currently, laparoscopy, a type of invasive surgery, is the gold standard to confirm endometriosis [5]. Additionally, the treatment for endometriosis is focused on symptomatic relief rather than curing the disease [6]. Pharmacotherapy and surgery, the most common means of endometriosis treatment at present, have high recurrence rates and adverse side effects [7]. Therefore, a noninvasive and sensitive examination to increase the likelihood of discovering endometriosis could help reduce the delay in diagnosis.

The pathogenesis of endometriosis is not currently known. The backflow of menstrual blood is the most commonly accepted hypothesis for the molecular mechanisms of endometriosis [8]. However, this theory has limitations and cannot explain all aspects of endometriosis. Epigenetic factors are also considered possible causes of endometriosis. N6-methyladenosine (m6A) methylation is one of the most frequent modifications of RNAs [9]. It is a reversible reaction, formed by methyltransferase complex (MTC) methylation of the sixth position N of adenine on mRNA [10], and demands a series of dynamic regulatory proteins encoded by writers, erasers, and readers to maintain the balance [11]. Previous studies suggested that m6A methylation is related to the proliferation, differentiation, invasion, and other biological behaviors of tumors, while abnormal m6A regulators have been recognized as new drug targets [12,13]. Notably, endometriosis is a benign disease but has several malignant traits, such as invasion, migration, reduced apoptosis, and defective differentiation [14]. Hence, m6A methylation might also be involved in the pathogenesis and development of endometriosis. Recent studies have proved that m6A methylation transferase methyltransferase like 3 (*METTL3*) might play a key role in the development of endometriosis [15,16,17], while the other two m6A regulators, *FTO* and *IGF2BP2*, have also been reported to participate [18,19,20]. However, the studies on the role of other m6A regulators in endometriosis are few and the underlying molecular mechanism is still unclear.

In our study, we comprehensively confirmed the role of m6A regulators in the prediction and subtype classification of endometriosis based on the GSE6364 dataset from the Gene Expression Omnibus (GEO) database. We constructed three distinct m6A patterns, among which there was a subtype highly consistent with significant low expression of *ESR1* and *PGR*, suggesting that the m6A pattern might be highly linked to endometriosis and could help to predict the occurrence of endometriosis and guide timely treatment.

## 2. Materials and Methods

### 2.1. Data Acquisition

**Figure 1:**
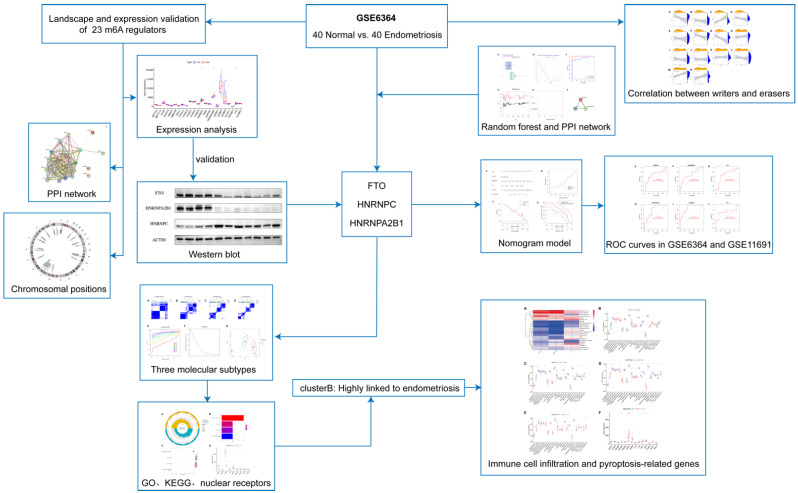
The work flow of our study. The work flow showed how we designed and conducted our study.

We drew a workflow illustration for our study (Figure 1). The GSE6364 dataset, consisting of 40 women with normal endometrial pathology and 40 women with laparoscopy-proven moderate-to-severe stage endometriosis, was downloaded from the GEO database [21]. In our study, a total of 23 m6A regulators were selected from the dataset by exploring significant m6A regulators utilizing analysis of endometriosis patients and non-endometriosis women. The twenty-three regulators included fourteen readers (*IGFBP1*, *IGFBP2*, *IGFBP3*, *YTHDC1*, *YTHDC2*, *YTHDF1*, *YTHDF2*, *YTHDF3*, *HNRNPA2B1*, *HNRNPC*, *FMR1*, *LRPPRC*, *ELAVL1*, and *IGF2BP1*), seven writers (*METTL3*, *METTL14*, *RBM15*, *RBM15B WTAP*, *ZC3H13*, and *CBLL1*), and two erasers (*ALKBH5* and *FTO*). The validation dataset (GSE11691) included nine normal endometria and nine endometriosis samples. The differential expression of the 23 m6A regulators between the normal group and endometriosis patients was analyzed through the “limma” R package, with the criterion of a *p*-value < 0.05, and was visualized via “reshape2” and “ggpubr” R packages. To identify and visualize the correlation between writers and erasers, we utilized “limma”, “ggplot2”, “ggpubr”, and “ggExtra” to evaluate the correlation coefficient R and *p*-value.

### 2.2. Construction of the PPI Network and the Random Forest Model

In order to predict the occurrence of endometriosis, the support vector machine (SVM) model and random forest (RF) were compared and evaluated by visualized receiver operating characteristic (ROC) curves and residuals, according to which we chose the RF model. During that process, R packages “caret”, “DALEX”, “ggplot2”, “kernlab”, and “pROC” were applied. RF is a machine learning model that has various applications in data analysis. For extracting candidate genes to predict endometriosis occurrence, the “RandomForest” package in R statistical software was utilized to implement the model. The average error rate of differentially expressed genes extracted above was calculated and utilized to choose an optimal count of variables. Then, we calculated the error rate of 1~500 trees to identify the optimal count with the best stability and the lowest error rate, based on which the random forest tree model was plotted. The feature importance scores were identified according to this RF model, and biomarkers whose importance scores were greater than 1.5 were considered the candidate genes of sarcopenia, according to the Gini coefficient.

We then evaluated and visualized the importance of the appropriate candidate m6A regulators through the RF model. Theprotein–protein interactions (PPIs) of both the candidate m6A regulators and all 23 m6A regulators were constructed by STRING (https://string-db.org/, accessed on 2 January 2022) [22]. We visualized the full PPI network with a minimum required interaction score of 0.4.

### 2.3. Construction of a Nomogram Model and an ROC Curve

According to the candidate m6A regulators, a nomogram model was plotted through the “rms” and “rmda” packages in R. To test if the model was accurate, the calibration curve and decision curve analysis (DCA) were constructed. In addition, a clinical impact curve was implemented to identify whether clinical decisions and diagnoses on the basis of the model could benefit endometriosis patients [23]. To further evaluate the accuracy of prediction based on candidate genes, we constructed ROC curves of each candidate gene both in GSE6364 and the validation dataset GSE11691 using the “pROC” package [24].

### 2.4. Identification of Subtypes on the Basis of Significant m6A Regulators

Consensus clustering was then utilized to explore both members and their subgroup numbers. We used this method to identify distinct m6A subtypes with a maxK, the consensus clustering coefficient, from 2 to 9, on the basis of the candidate m6A regulators through the “ConsensusClusterPlus” package [25].

### 2.5. Identification of DEGs among Distinct m6A Subtypes

We utilized the “limma” package [26] in R to extract differentially expressed genes (DEGs) with a screening criterion of *p* < 0.05 and applied principal component analysis (PCA) algorithms to distinguish m6A subtypes.

### 2.6. Functional and Pathway Enrichment Analysis of DEGs

The Kyoto Encyclopedia of Genes and Genomes (KEGG) pathway enrichment, as well as Gene Ontology (GO) functional enrichment, were applied to reveal the underlying molecular mechanism of the DEGs related to endometriosis. The “clusterProfiler” package [27] was utilized to identify the GO biological processes, which were shown in three characteristics, including cellular components (CCs), biological processes (BPs), and molecular functions (MFs). An enrichment circle diagram was used to visualize the enrichment analysis results. The KEGG is a database for gene function analysis [28]. We used the “clusterProfiler”, “org.Hs.eg.db”, “enrichplot”, and “ggplot” packages to perform and visualize pathway enrichment.

### 2.7. Identification of Immune Cell Infiltration

In order to identify the abundance of various immune cells in endometriosis samples, single-sample gene set enrichment analysis (ssGSEA) [29] was applied in our study. It was used to explore and obtain gene expression in endometriosis samples to identify the correlation between immune cells and genes. We also utilized ssGSEA to obtain the correlation between immune cell infiltration and candidate gene expression. We applied “reshape2”, “ggpubr”, “limma”, “GSEABase”, and “GSVA” R packages to demonstrate immune cell infiltration in three distinct subtypes, while the “limma”, “pheatmap”, “reshape2”, and “ggpubr” packages were utilized to analyze the correlation between candidate genes and immune infiltration, as well as the expression of pyroptosis-related genes in three patterns.

### 2.8. Western Blot

The study was approved by the Ethics Committee of Shanghai First Maternity and Infant Hospital (KS21198). Ovarian endometriosis tissues, as well as normal endometrium tissues, were collected, and then proteins were extracted through a RIPA lysis buffer (PC101, EpiZyme, Shanghai, China). The proteins (20 µg) were loaded and electrophoresed on SDS-polyacrylamide gels. A protein-free rapid-blocking buffer was used for blocking. Then, membranes were probed with primary antibodies against FTO (1:2000, Abclonal, Wuhan, China), HNRNPC (1:1000, Abclonal, Wuhan, China), or HNRNPA2B1 (1:1000, Abclonal, Wuhan, China) overnight. The internal control in our study was the anti-β-Actin antibody (1:5000, Abclonal, Wuhan, China). Then, the membranes were incubated with secondary antibodies and the target protein bands were visualized. We used Image J software (version Java 1.8.0_172, Wayne Rasband, National Institutes of Health, Bethesda, MD, USA) to evaluate the protein band densities.

### 2.9. Statistical Analysis

Pearson’s correlation analysis was performed to identify the correlation between erasers and writers, while the Kruskal–Wallis test was utilized to explore differences among the groups. Two-tailed tests were used for all parametric analyses, with statistical significance set at *p* < 0.05. All of the statistical calculations were conducted utilizing R (version 4.1.1).

## 3. Results

### 3.1. Landscape and Expression Validation of 23 m6A Regulators in Endometriosis

**Figure 2:**
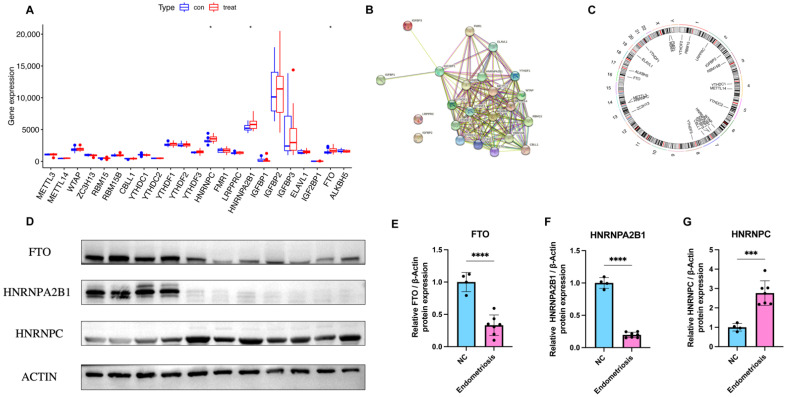
Landscape and expression validation of the 23 m6A regulators in endometriosis. (**A**) Differential expression levels of the 23 m6A regulators between non-endometriosis and endometriosis patients. (**B**) PPI network of the 23 m6A regulators. (**C**) Chromosomal positions of the 23 m6A regulators. (**D**–**G**) The protein expression of three significant m6A regulators in control samples (*n* = 4) and endometriosis samples (*n* = 7). * *p* < 0.05, *** *p* < 0.001, and **** *p* < 0.0001.

We show the landscape of the 23 m6A regulators in endometriosis and the validation of differentially expressed genes in Figure 2. Three m6A regulators (*HNRNPC*, *HNRNPA2B1*, and *FTO*) were identified as being significantly overexpressed in endometriosis patients and were visualized utilizing a boxplot (Figure 2A). To validate the expression level of heterogeneous nuclear ribonucleoprotein C (*HNRNPC*), heterogeneous nuclear ribonucleoprotein A2/B1 (*HNRNPA2B1*), and fat mass and obesity-associated protein (*FTO*), we applied a Western blot, and the results indicate that *HNRNPC* was significantly overexpressed in the endometriosis group while the other two genes were obviously downregulated at the protein level (Figure 2D–G). STRING was utilized to construct the PPI network (Figure 2B). There were 23 nodes and 150 edges in the PPI network, with a PPI enrichment *p*-value < 1.0 × 10^−16^ and an average local clustering coefficient of 0.83. The “RCircos” package was applied to identify the chromosomal positions of the 23 m6A regulators and the results are visualized in Figure 2C, from which we found that *FMR1* and *RBMX* were on the sex chromosome, while other regulators were on the autosomes. Chromosome 7 had the most regulators, including *WTAP*, *HNRNPA2B1*, *IGFBP1*, *IGFBP3*, and *CBLL1*, while there was no regulator on chromosomes 9, 10, 11, 12, 15, 18, 21, 22, or Y.

### 3.2. Correlation between Erasers and Writers in Endometriosis

**Figure 3:**
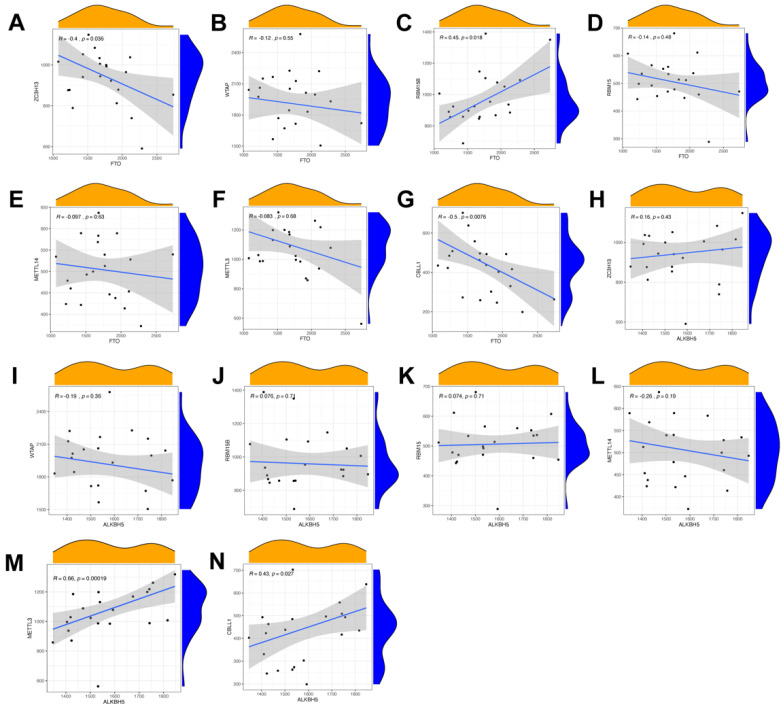
Correlation between erasers and writers in endometriosis. (**A**–**N**). Writer genes: *WTAP*, *ZC3H13*, *METTL3*, *RBM15*, *RBM15B*, *METTL14*, and *CBLL1*; eraser genes: *ALKBH5* and *FTO*.

To elucidate the relationship of expression levels between writer genes (*RBM15*, *RBM15B*, *METTL14*, *METTL3*, *ZC3H13*, *WTAP*, and *CBLL1*) and eraser genes (*ALKBH5* and *FTO*), we utilized Pearson’s correlation analysis. The correlation between each writer gene and eraser gene is shown in Figure 3. We considered the correlation coefficient R > 0.4 and *p* < 0.05 to indicate a significant relationship. The expression levels of *CBLL1* and *ZC3H13* had a significant negative correlation with *FTO* (Figure 3A,G), while RBM15B had a positive correlation with *FTO* (Figure 3C). Endometriosis patients with a high expression of *CBLL1* and *METTL3* showed a notably high expression level of *ALKBH5* (Figure 3M,N). There were no significant correlations between other writers and erasers. In conclusion, we discovered that there were various correlations between different writers and erasers.

### 3.3. Construction of the RF Model of m6A Regulators and the PPI Network of Candidate Genes

**Figure 4:**
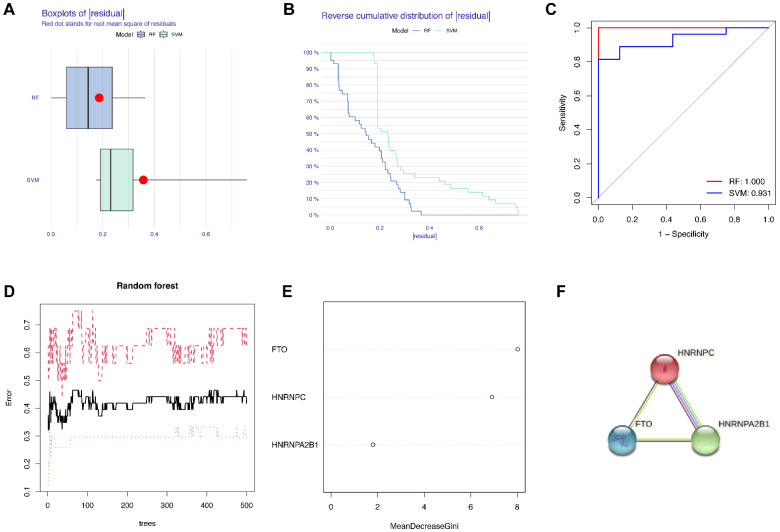
Construction of the RF model of m6A regulators and the PPI network of candidate genes. (**A**) Boxplots of residual. (**B**) Reverse cumulative distribution of residual. (**C**) The ROC curve indicated the accuracy of the SVM and RF models. (**D**) The tree numbers of random forest. (**E**) The importance of significant genes. (**F**) The PPI network of significant genes.

In order to search for candidate diagnostic m6A regulators, we constructed an RF model. The process of selecting the RF and SVM models and the key candidate genes discovered are shown in Figure 4. To evaluate these two models, we calculated and visualized the residual by establishing “Boxplots of residual” and “Reverse cumulative distribution of residual” (Figure 4A,B). The results show that the residual of the random forest model was minimal, indicating that the RF model is better. In addition, we plotted an ROC curve (Figure 4C) to compare the RF model with the SVM model, and the area under the ROC curve (AUC) value indicated that the RF model was also more accurate. Hence, we chose the RF model to predict the diagnostic genes of endometriosis. We input the three candidate biomarkers into the RF and set the optimal parameter, followed by identifying all possible counts in the one to three variables with recurrent RF classification. Subsequently, the average error rate was determined, and based on both the decision trees and the model error rate, the stable error of the RF model could be identified (Figure 4D). Then, we used the Gini coefficient method to identify the importance scores of three variables, which helped to decrease the accuracy and mean square error while constructing the RF model. The importance scores of candidate genes are visualized in Figure 4E, from which we found that all the candidate biomarkers had an importance score of greater than 1.5, so they could all be considered potential candidate biomarkers for endometriosis. The PPI network of these three candidate genes was established through STRING (Figure 4F). In this PPI network, there were three nodes and three edges, with a PPI enrichment *p*-value < 0.05 and an average local clustering coefficient of 1.

### 3.4. Construction of the Nomogram Model and the ROC Curve

**Figure 5:**
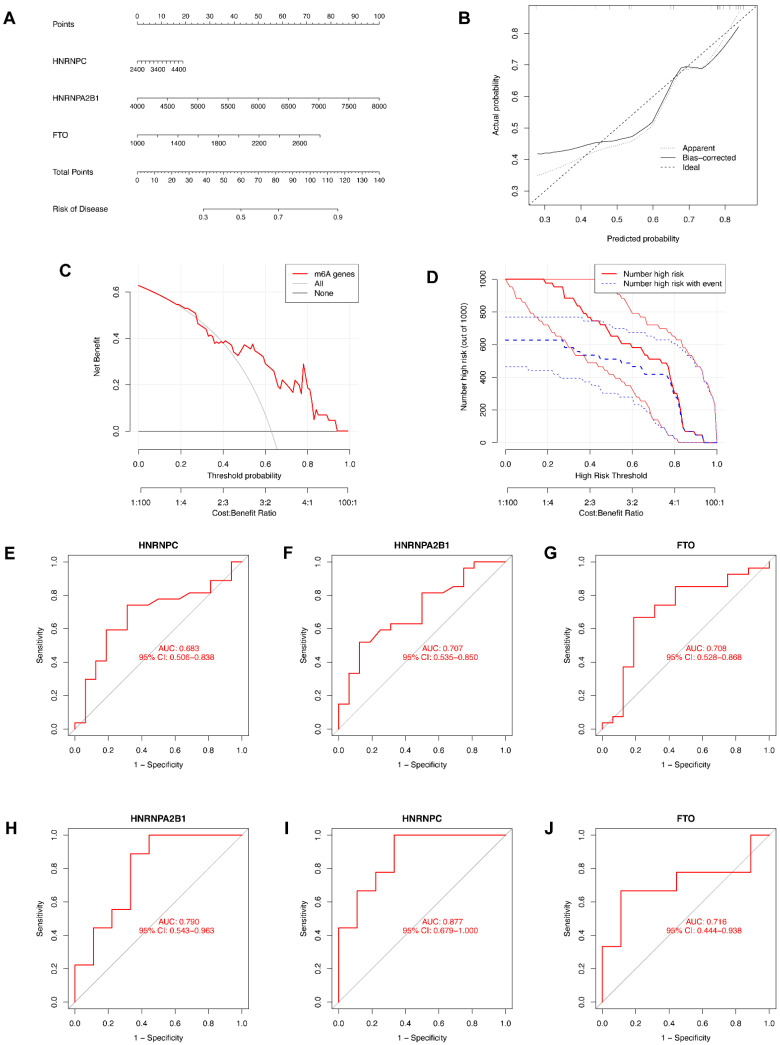
Construction of the nomogram model and the ROC curve. (**A**) The nomogram model based on the candidate genes. (**B**) The calibration curve of the nomogram model. (**C**) The DCA curve of the nomogram model. (**D**) The clinical impact curve of the nomogram model. (**E**–**G**) ROC curves of candidate genes based on GSE6364. (**H**–**J**) ROC curves of candidate genes based on GSE11691 for validation.

We then constructed a nomogram to identify the prevalence of endometriosis and identified the predictive ability of each candidate biomarker via ROC curves, both of which are visualized in Figure 5. The nomogram model based on the candidate genes was constructed by the “rms” package in R software (Figure 5A). The nomogram model was reliable and predictable according to calibration curves (Figure 5B). In the DCA curve (Figure 5C), the red line was above the black and gray ones in the majority of areas, which indicates that making decisions based on the nomogram model would be beneficial for endometriosis patients. The predictive ability of the nomogram model was proved to be notable through the clinical impact curve (Figure 5D). To further test the predictive power of the candidate genes, we then constructed the ROC curves based on GSE6364 and revealed the diagnostic value of candidate genes according to the AUC (Figure 5E–G). Another series, GSE11691, was utilized as a validation dataset, based on which we also constructed ROC curves and obtained the AUC (Figure 5H–J). All of the AUCs were greater than 0.65, while the AUC of *HNRNPC* in the validation dataset was greater than 0.8, indicating that all three candidate genes were of significance in the diagnosis of endometriosis.

### 3.5. Identification of Three Molecular Subtypes with Unsupervised Clustering of Significant m6A Regulators

**Figure 6:**
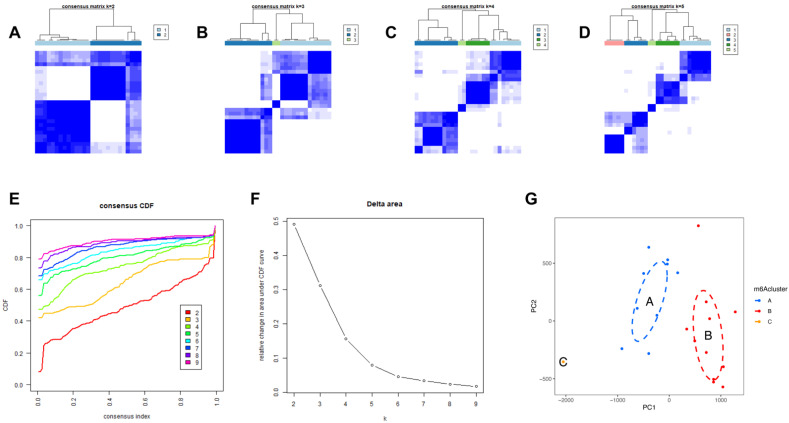
Identification of three molecular subtypes through unsupervised clustering of significant m6A regulators. (**A**–**D**) Distinct m6A patterns based on the candidate genes. (**E**) The CDF graph. (**F**) Relative change of the area under the CDF curve (k  =  2–9). (**G**) Principal component analysis of the candidate genes in the three subtypes.

**Figure 7:**
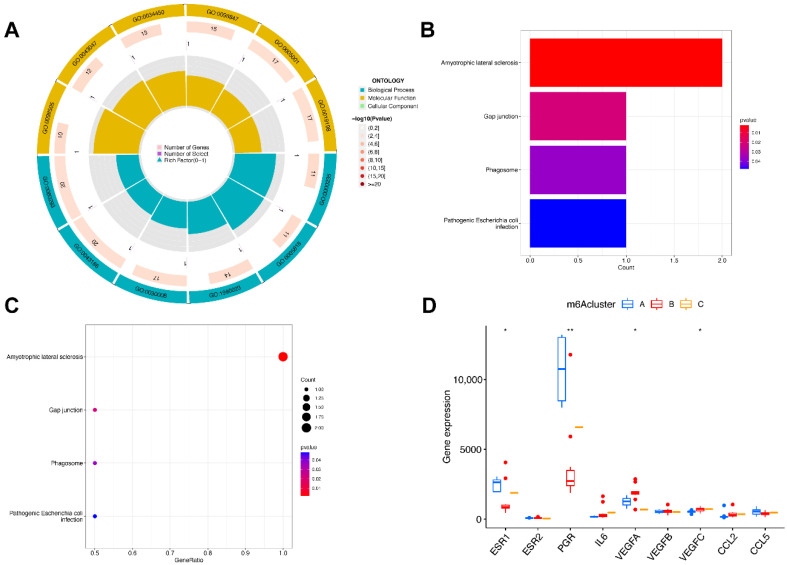
Pathway and functional enrichment of DEGs and the expression level of endometriosis biomarkers in three clusters. (**A**) GO functional enrichment. (**B**,**C**) The KEGG pathway enrichment. (**D**) The differential expression of endometriosis biomarkers in three subtypes. * *p* < 0.05 and ** *p* < 0.01.

We then identified molecular subtypes according to candidate genes, based on which we subsequently applied functional enrichment. We used the unsupervised clustering method to distinguish distinct m6A patterns on the basis of three significant m6A regulators with the “ConsensusClusterPlus” package (Figure 6A–D). The area under the CDF curve indicated a stable partition of the samples starting at the four clusters (Figure 6E,F), and the PCA showed a near-perfect stable partitioning of the samples in the three clusters (Figure 6G). The three m6A patterns could be clearly identified through three significant m6A regulators, according to PCA. After assessing the results, including the area under the CDF curve, PCA, and the practical clinical significance, the three-cluster solution was selected. ClusterA contained thirteen cases, clusterB contained twelve cases, and clusterC contained two cases. The exact group classification and candidate gene expression in the three distinct m6A patterns are shown in Table S1. With the criteria of logFC = 1 and adj.P.Val = 0.05, we made a comparison between each group to extract the intersection of DEGs (Figure S1). There were 3435 DEGs between clusterA and clusterB, and there were 103 DEGs between clusterC and clusterB, while there were only 47 DEGs between clusterA and clusterC. The intersectional genes of DEGs were *HNRNPA2B1*, *HMGN5*, *HIST2H2BE*, *MAP9*, *TRAPPC6B*, *TUBB2A*, *PTPRG*, *ZFYVE21*, *PELI1*, and *TDRD9*. Hence, we then selected these ten m6A-related DEGs among the three m6A patterns and applied GO functional enrichment (Figure 7A) and the KEGG pathway enrichment (Figure 7B,C). We found that the genes were equally enriched. Each GO had one selected gene, except the microtubule, which had two genes, *MAP9* and *TUBB2A*. The results of the GO enrichment analysis are provided in Table S2. The DEGs mainly participated in four pathways in our study, including amyotrophic lateral sclerosis, gap junction, phagosome, and pathogenic escherichia coli infection.

We then analyzed the correlation between the nuclear receptors and the three m6A patterns (Figure 7D). The results illustrate that clusterB was correlated with a significantly lower expression of *ESR1* and *PGR* and a higher expression of *VEGF*, which revealed that the cluster might have a correlation with endometriosis.

### 3.6. Immune Cell Infiltration and Pyroptosis-Related Genes of m6A Patterns in Endometriosis

**Figure 8:**
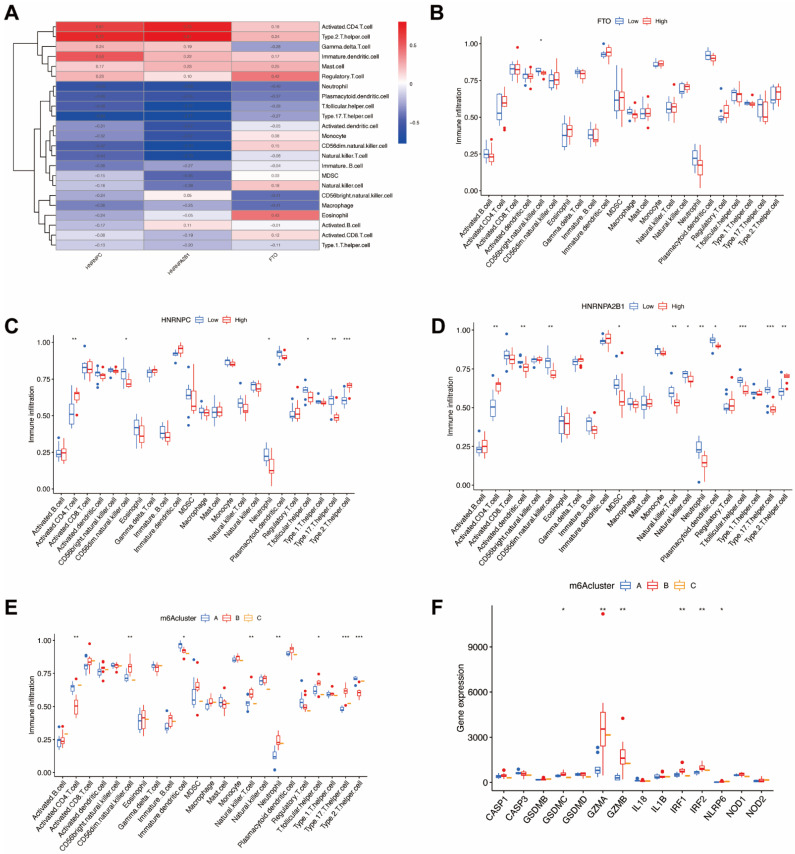
Immune cell infiltration and pyroptosis-related genes of m6A patterns in endometriosis. (**A**) The relationship between 23 kinds of immune cells and the candidate genes. (**B**) Variation and difference in the immune cell infiltration between low and high *FTO* expression groups. (**C**) Difference and variation in the immune cell infiltration between low and high *HNRNPC* expression groups. (**D**) Difference and variation in the immune cell infiltration between low and high *HNRNPA2B1* expression groups. (**E**) The correlation between the three m6A patterns and the infiltration of 23 immune cells (**F**) The relationship between pyroptosis-related genes and the three m6A patterns. * *p* < 0.05, ** *p* < 0.01, and *** *p* < 0.001.

In order to demonstrate the occurrence and quantity of immune cells in endometriosis samples, we utilized ssGSEA to evaluate the relationship between 23 kinds of immune cells (Figure 8A) and the significant m6A regulators. Moreover, we also identified the immune cell infiltration between individual patients with low and highly significant m6A regulator expressions (Figure 8B–D). The results indicate that high *FTO* infiltration leads to decreased immune cell infiltration. Both high *HNRNPC* and *HNRNPA2B1* infiltration decreased immune cell infiltration, except for type 2 T-helper cells and activated CD4 T cells.

We then analyzed the correlation between three m6A patterns and various immune cell infiltration types (Figure 8E) and discovered that clusterB was correlated with high type 17 T-helper cell (Th17) infiltration and high neutrophils infiltration, which indicates that clusterB may be related to endometriosis. In order to further demonstrate the correlation between endometriosis and m6A patterns, we revealed and elucidated the relationship between m6A patterns and pyroptosis-related genes (Figure 8F). The expression levels of pyroptosis-related genes were significantly higher in clusterB, which also indicated that clusterB was highly linked to endometriosis because of the overexpression of inflammatory genes.

## 4. Discussion

Endometriosis is a common and complex inflammatory disorder that affects nearly 10% of reproductive-age women. Despite its prevalence, however, this disease lacks accurate diagnosis and prompt therapy [30]. The most abundant mRNA chemical modification, N6-methyladenosine, is proven to be involved in numerous biological processes and molecular modification [31,32]. N6-methyladenosine participates in biological processes dynamically and reversibly via regulating methyltransferases (writers), demethylases (erasers), and m6A-binding proteins (readers). Recent years have seen increasing evidence of the relationship between m6A and female reproduction diseases, including endometriosis, reproductive system tumors, premature ovarian failure, polycystic ovary syndrome, and adenomyosis [20]. This research has demonstrated that some m6A regulators, such as *METTL3*, *FTO*, and *IGF2BP2*, participate in the process and development of endometriosis, while there are few studies on other m6A regulators. In our study, we aimed to show a landscape of m6A regulators in endometriosis, followed by identifying and demonstrating the characteristics of significant biomarkers to provide further guidance for the diagnosis and clinical treatment of endometriosis.

Three significant m6A regulators were identified among twenty-three m6A regulators between endometriosis and non-endometriosis groups. Though the expression of *HNRNPA2B1* and *FTO* showed opposite trends between bioinformatic analysis and validation, they demonstrated a significant expression change in the endometriosis group at the protein level. The limited number of samples in the datasets might account for this inconformity. We then analyzed the correlation between writers and erasers in endometriosis and utilized an RF model to visualize the importance of candidate genes and evaluate the occurrence of endometriosis. In order to evaluate the risk of endometriosis, we implemented a nomogram model based on candidate genes, and the model proved to be beneficial for patients through the DCA curve. ROC curves were also plotted based on the dataset used in our study and the validation dataset GSE11691 to predict the occurrence of endometriosis and validate the accuracy of the model. Three distinguishable subtypes were then constructed according to the significant m6A regulators. ClusterB was revealed to be associated with significantly overexpressed *VEGFA* and *VEGFC* and, notably, downregulated *ESR1* and *PGR*, which are characteristic biomarkers of endometriosis [33], indicating that clusterB might be highly linked to endometriosis [30]. The regulation of these genes, especially *ESR1* [34] and *PGR* [35], is known to induce estrogen-dependent inflammation and progesterone resistance in endometriosis patients. Additionally, the functional enrichment analysis and pathway enrichment analysis based on DEGs among three molecular subtypes implied that cellular components and molecular functions play a key role in endometriosis, and that four main pathways (amyotrophic lateral sclerosis, gap junction, phagosome, and pathogenic escherichia coli infection) are enriched and highly linked to the disease.

*FTO* (fat mass and obesity-associated protein) is involved in the control of energy expenditure and adipogenesis, and is associated with oxidative stress and mitochondrial biogenesis [36]. Several reports have indicated that the expression change of *FTO* is associated with the occurrence of various cancers through stimulating cellular metabolism [37]. *HNRNPC* and *HNRNPA2B1* are two members of the hnRNPs family. Both are associated with numerous molecular processes involving pre-mRNA splicing. It was reported that *HNRNPA2B1* has a positive correlation with *ESR1* and *PGR* [12], which is consistent with the results of our study. The expression of *HNRNPA2B1*, *ESR1*, and *PGR* in cluster B was downregulated. Until now, the role of *HNRNPC* in endometriosis remained unclear, while the protein levels of *HNRNPA2B1* were revealed to be downregulated in endometriosis [38]. The lower expression levels of *HNRNPA2B1* are correlated with higher levels of Th17 cells and neutrophil infiltration, which have been proved to be increased in endometriosis [39].

Neutrophils and HNP 1–3 (human neutrophil peptides 1, 2, and 3) have been reported to be correlated with the immunopathogenesis of endometriosis at an early stage through inducing angiogenesis and modulating the local inflammatory environment [40,41]. Elevated circulating plasma neutrophil levels could also indicate an inflammatory status [42]. The alteration of regulatory T cells (Tregs) and Th17 cells might contribute to the presence of ectopic endometrial lesion implantation and impact the development of endometriosis toward an advanced stage [43]. A reduction in the Treg/Th17 ratio with lower Tregs and higher Th17, indicating an imbalance in the immune microenvironment and systemic inflammation, was discovered in patients with endometriosis [44]. In our study, immune infiltration of clusterB showed an elevated level of Th17 cells and a reduced level of Tregs, which is consistent with other findings on endometriosis. Probably on account of the detection methods or the limited number of samples used, however, the reduction in Tregs was not significant. Pyroptosis is a new inflammatory form of programmed cell death that is essential for immunity. It was demonstrated that pyroptotic mediators may have an essential influence on the immune cells of adaptive immunity through various mechanisms and can also be reciprocally influenced by adaptive immune cells [45]. Inflammation is considered to have an essential role in endometriosis through modulating angiogenesis, implantation, and proliferation [46]. Both pyroptosis and its correlative inflammasomes have been shown to play an essential role in endometriosis [47]. In this study, most of the pyroptosis-related genes were evaluated and found to be significantly overexpressed in clusterB, which indicates that clusterB is highly linked to endometriosis.

The candidate m6A regulators also have clinical implications in terms of diagnosis. We have identified and validated the expression of candidate biomarkers in endometrium tissue, so it might be possible to obtain endometria from menstruation blood and detect the expression of significant biomarkers to achieve an early endometriosis diagnosis [48]. Additionally, detecting the expression of candidate biomarkers could be a supplementary method in laparoscopy, possibly improving diagnosis for endometriosis patients. However, this method might not yet be suitable as a routine test for endometriosis patients, as the data for bioinformatic analysis were obtained from publicly available databases and the number of samples was relatively limited. More biological and clinical studies with a larger number of samples and examining more clinical characteristics of endometriosis patients should be carried out to further validate our conclusions. Additionally, there were other limitations in our research. Firstly, the pathogenesis of endometriosis is multidimensional, so discussing it only from the aspect of N6-methyladenosine methylation or immune infiltration may not provide a sufficiently comprehensive understanding of this process. Secondly, though we validated the expression of candidate m6A genes at the protein level via Western blotting, more biological studies including in vivo and in vitro experiments should be performed, and more prospective and multicenter studies are needed to evaluate the potential clinical applications of molecular signatures, as well as to elucidate the specific molecular mechanisms of candidate biomarkers.

## 5. Conclusions

In conclusion, our study aimed to evaluate the potential function of m6A regulators and identify candidate biomarkers for diagnostic and prognostic treatments in endometriosis patients. We extracted three significant m6A regulators, based on which we constructed a nomogram model that could accurately predict the prevalence of endometriosis. We further identified three subtypes based on the three candidate genes. ClusterB demonstrated high levels of Th17 cells, neutrophil infiltration, and overexpressed pyroptosis-related genes, which, together with the change in endometriosis biomarkers, highly indicates a correlation with endometriosis.

## Data Availability

The datasets generated and analyzed during the current study are available in the Gene Expression Omnibus (GEO) database, https://www.ncbi.nlm.nih.gov/geo/query/acc.cgi?acc=GSE6364 (accessed on 1 January 2022), https://www.ncbi.nlm.nih.gov/geo/query/acc.cgi?acc=GSE11691 (accessed on 1 January 2022).

## Supplementary Materials

The following supporting information can be downloaded at https://www.mdpi.com/article/10.3390/genes14010086/s1. R code: The codes used in our research via R software. Figure S1: The intersection of DEGs; Table S1: Candidate gene expression and group classification in 3 distinct m6A patterns; Table S2: The enriched GO terms of differentially expressed genes.
