# Supplementary material for: Identification and Validation of Three m6A Regulators: FTO, HNRNPC, and HNRNPA2B1 as Potential Biomarkers for Endometriosis
Source: Genes (Basel). 2022 Dec 28;14(1):86. doi: 10.3390/genes14010086 (PMC9858668; doi:10.3390/genes14010086)
Supplement: Supplementary file 1 [file genes-14-00086-s001.zip › Table S1.pdf]

Table S1. Candidate gene expression and group classification in 3 distinct m6A patterns.

ID	HNRNPC	HNRNPA2B1	FTO	m6Acluster
GSM150195_treat	3670.296	6036.206	1676.975	A
GSM150194_treat	4142.779	6182.694	1770.507	A
GSM150193_treat	3865.898	5801.169	2112.648	A
GSM150192_treat	3849.21	5590.758	1942.794	A
GSM150191_treat	3523.232	5326.378	2735.089	B
GSM150190_treat	4457.508	5993.68	1767.169	A
GSM150219_treat	3636.539	5252.191	1283.465	B
GSM150218_treat	3277.48	4955.735	1076.968	B
GSM150217_treat	3138.834	5004.853	1238.972	B
GSM150216_treat	3029.223	5216.749	1681.033	B
GSM150215_treat	3409.78	5133.821	1919.415	B
GSM150214_treat	2793.871	4745.617	2283.633	B
GSM150213_treat	3274.605	5095.796	1836.448	B
GSM150212_treat	3058.305	4845.218	1430.242	B
GSM150211_treat	3033.172	4845.218	1217.514	B
GSM150207_treat	3225.981	6320.337	1670.933	A
GSM150206_treat	2905.7	5680.125	2134.23	B
GSM150205_treat	3547.242	6761.105	1512.38	A
GSM150204_treat	3877.755	5801.169	2052.449	A
GSM150203_treat	3673.836	7899.378	1431.366	C
GSM150202_treat	3905.041	6362.645	1602.733	A
GSM150207_treat.1	3225.981	6320.337	1670.933	A
GSM150206_treat.1	2905.7	5680.125	2134.23	B
GSM150205_treat.1	3547.242	6761.105	1512.38	A
GSM150204_treat.1	3877.755	5801.169	2052.449	A
GSM150203_treat.1	3673.836	7899.378	1431.366	C
GSM150202_treat.1	3905.041	6362.645	1602.733	A
