# Supplementary material for: Identification and Validation of Three m6A Regulators: FTO, HNRNPC, and HNRNPA2B1 as Potential Biomarkers for Endometriosis
Source: Genes (Basel). 2022 Dec 28;14(1):86. doi: 10.3390/genes14010086 (PMC9858668; doi:10.3390/genes14010086)
Supplement: Supplementary file 1 [file genes-14-00086-s001.zip › Table S2.pdf]

Table S2. The enriched GO terms of differentially expressed genes.

ID	Category	GO Name	P.value	geneID
GO:0000235	CC	astral microtubule	0.00505359	MAP9
GO:0005818	CC	aster	0.00505359	MAP9
GO:1990023	CC	mitotic spindle midzone	0.00642789	MAP9
GO:0030008	CC	TRAPP complex	0.00780051	TRAPPC6B
GO:0043186	CC	P granule	0.00917144	TDRD9
GO:0060293	CC	germ plasm	0.00917144	TDRD9
GO:0045495	CC	pole plasm	0.00962805	TDRD9
GO:0005874	CC	microtubule	0.01561641	MAP9/TUBB2A
GO:0051233	CC	spindle midzone	0.01645468	MAP9
GO:0099023	CC	vesicle tethering complex	0.02818849	TRAPPC6B
GO:0005876	CC	spindle microtubule	0.03266825	MAP9
GO:0005801	CC	cis-Golgi network	0.03311522	TRAPPC6B
GO:0005881	CC	cytoplasmic microtubule	0.0340086	MAP9
GO:0015030	CC	Cajal body	0.03534729	HNRNPA2B1
GO:0071013	CC	catalytic step 2 spliceosome	0.03935348	HNRNPA2B1
GO:0016363	CC	nuclear matrix	0.04996446	HNRNPA2B1
GO:0098505	MF	G-rich strand telomeric DNA binding	0.00489023	HNRNPA2B1
GO:0043047	MF	single-stranded telomeric DNA binding	0.00586572	HNRNPA2B1
GO:0034450	MF	ubiquitin-ubiquitin ligase activity	0.00635315	PELI1
GO:0098847	MF	sequence-specific single stranded DNA binding	0.00732737	HNRNPA2B1
GO:0005001	MF	transmembrane receptor protein tyrosine phosphatase activity	0.00830073	PTPRG
GO:0019198	MF	transmembrane receptor protein phosphatase activity	0.00830073	PTPRG
GO:0035198	MF	miRNA binding	0.01654017	HNRNPA2B1
GO:0042162	MF	telomeric DNA binding	0.0175055	HNRNPA2B1
GO:0031492	MF	nucleosomal DNA binding	0.01943364	HMGN5
GO:0061980	MF	regulatory RNA binding	0.02231954	HNRNPA2B1
