# Supplementary figures and images for: Identification and Validation of Three m6A Regulators: FTO, HNRNPC, and HNRNPA2B1 as Potential Biomarkers for Endometriosis
Source: Genes (Basel). 2022 Dec 28;14(1):86. doi: 10.3390/genes14010086 (PMC9858668; doi:10.3390/genes14010086)

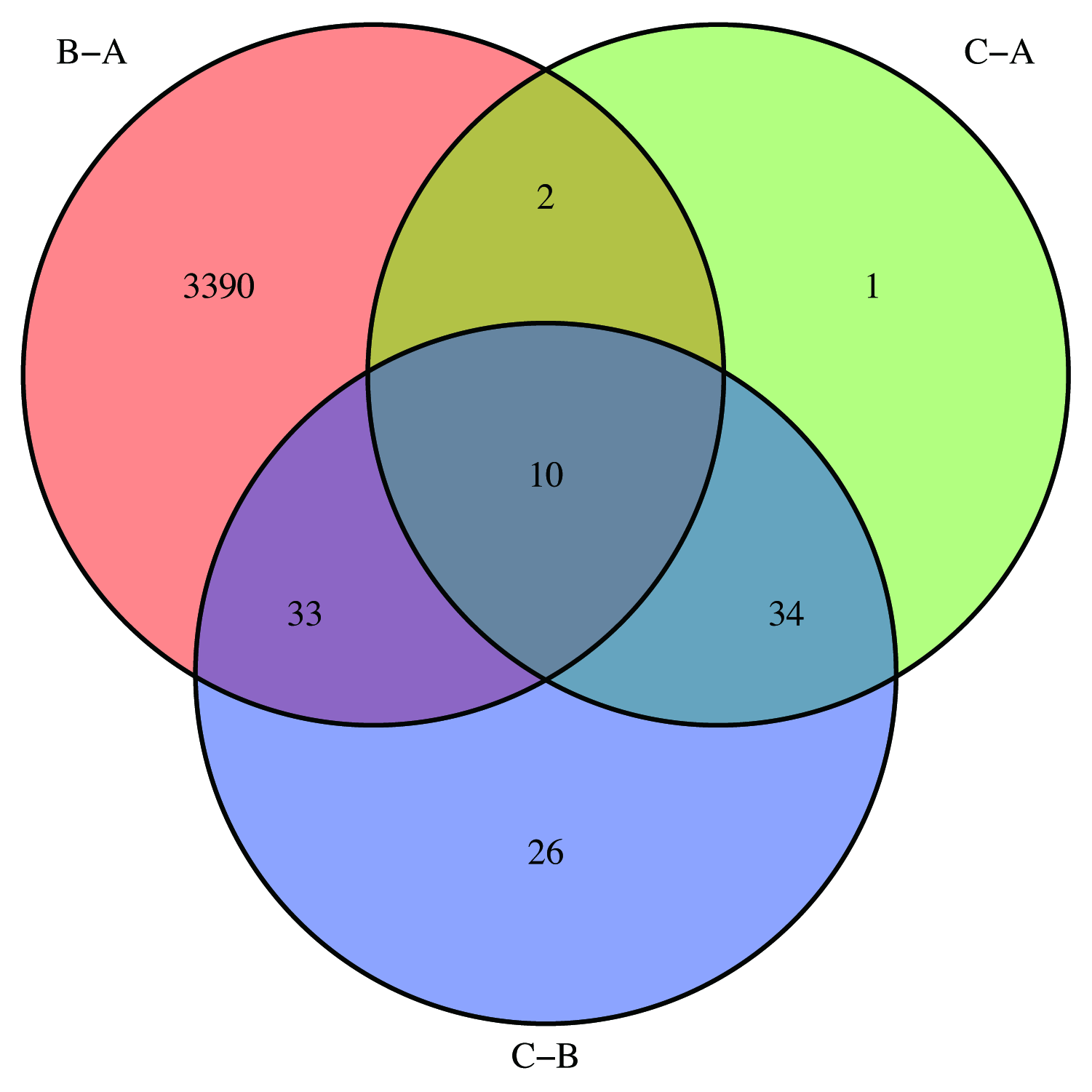

Supplement: Supplementary file 1 [file genes-14-00086-s001.zip › Figure S1.tif]
